# The Effect of Combined Processing on Residual Stresses in the Surface Layer of Power Plant Parts

**DOI:** 10.3390/ma15020420

**Published:** 2022-01-06

**Authors:** Anna Yakovleva, Margarita Isaenkova, Roman Minushkin

**Affiliations:** 1 Department of Machine Building, Bauman Moscow State Technical University, 2nd Baumanskaya St. 5, 105005 Moscow, Russia; 2 Department of Materials Science, National Research Nuclear University MEPhI, Kashirskoe Shosse 31, 115409 Moscow, Russia; isamarg@mail.ru (M.I.); minushkinroman@yandex.ru (R.M.)

**Keywords:** combined processing, residual stresses, power plants, technological heredity

## Abstract

The purpose of this research was to analyze the change in residual stresses in the surface layer of steel samples taking into account the technological heredity effect on the value and sign of residual stresses. An installation of combined processing was developed. Combined processing consists of sequentially performing electromechanical processing and diamond smoothing. All areas of the samples were studied—after machining (i.e., in the initial state), after electromechanical processing, and after diamond smoothing. The research shows that the sign and value of residual stresses are significantly affected by the combined processing modes. The main parameters of the surface layer are formed at the final stage of the combined processing–diamond smoothing. This paper gives recommendations on the use of combined processing for power plant parts.

## 1. Introduction

Residual stresses determine the quality and operational characteristics of hydropower plants [1,2,3,4,5,6,7,8]. The level of residual stresses is in many cases an important parameter that determines the quality of these mechanisms. Residual stresses can play both a positive and a negative role in changing the wear resistance of products, thereby determining their performance during operation.

Significant residual stresses can occur after machining—lathe turning, milling, grinding—which act only in the surface layer with a depth of a few tenths of a millimeter. On the other hand, operating experience shows that it is these stresses that can affect the wear resistance and strength of the part, especially under the action of alternating stresses. In the surface layer of the metal there are always various defects that serve as crack nuclei; therefore, the reliability of the parts is determined mainly by the intensity of the work of crack origins.

Compressive residual stresses formed on the surfaces of parts by various technological methods prevent the initiation and propagation of fatigue microcracks, increasing their performance. Therefore, for hydropower plant parts operating under sliding friction conditions, especially in cases of fatigue and abrasive wear during operation, it is important to create a surface layer with compressive residual stresses [4,6,7,8,9,10,11,12].

The operational properties of parts depend on the state of the surface layer. It was found that the manufacture of parts from the same material, but with different technologies and different processing modes, leads to an abrupt change in the properties of the surface layer, while the durability of such parts is different.

The studies of technology are usually limited to certain operations.

When using high-intensity energy flow influences, however, it is necessary to comprehensively study the accuracy and physico-mechanical properties, taking into account the effect of technological and operational heredity. This means that all operations and their technological transitions, as well as the stages of operation, should not be considered in isolation, but in a coordinated fashion, since the characteristics of the processed surfaces are formed by the whole set of technological effects and change during a part’s operation.

Technological heredity is manifested particularly vividly in combined processing methods, when technological factors sequentially and simultaneously affect the work part. Moreover, heredity not only has an effect immediately after the finishing operations of the technological process, but can also occur during the operation as a result of the effect of certain surface quality parameters created in the surface layer of the part during its previous processing [1,2,9,10,11,12,13,14,15,16,17,18].

From the standpoint of technological and operational heredity, it is possible to establish a connection between technological operations and transitions, including the cases where combined methods of machine parts are applied [3,13].

In this work, we will study the stresses of the first kind after machining and after combined processing, as well as the hereditary effect of the combined processing modes on the value and sign of residual stresses. Stresses of the first kind are the most significant in terms of practical application [12].

It should be noted that technological processes are associated with the formation of specific deviations and with the operational properties of machine parts. This connection constitutes the theory of technological heredity [14]. The theory of technological heredity should be used to analyze the influence of processing methods and processing modes on the production process [15]. Properties that positively affect the quality of the surface layer should be preserved during operation, while those that negatively affect the quality of the surface should be eliminated in the manufacturing process of the part.

## 2. Description of the Experiment

**Figure 1:**
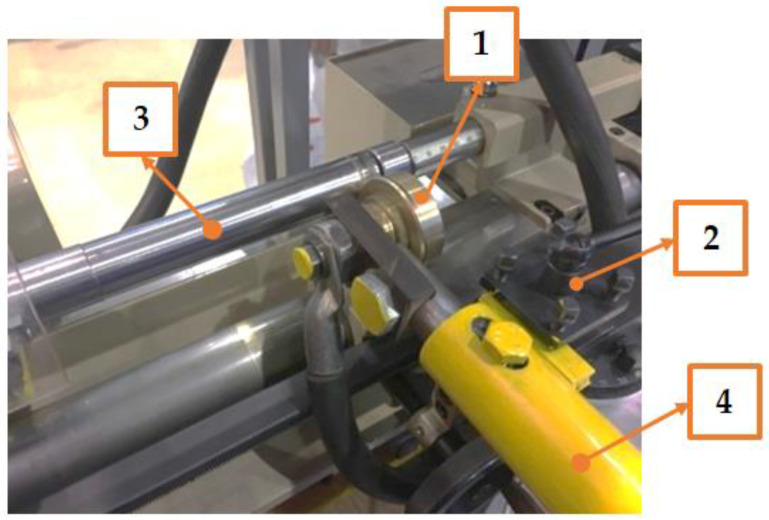
The Start bench lathe trial plant: 1—roller electrode, 2—power supply system, 3—sample, 4—tool holder.

The combined processing method consists of the sequential use of electromechanical processing (EMP) and diamond smoothing [17,18]. We developed a trial plant consisting of the Start bench lathe, a power source, a quench, a current supply system, and a quenching area cooling system, in which combined processing modes were tested [19,20] (Figure 1). First, the samples were turned on a desktop lathe “Start” (Figure 1): the depth of cut was 1 mm, the part peripheral speed was 26 m/min, the feed rate was 0.3 mm/rev, and the number of revolutions of the machine spindle was 700 rev/min.

**Figure 2:**
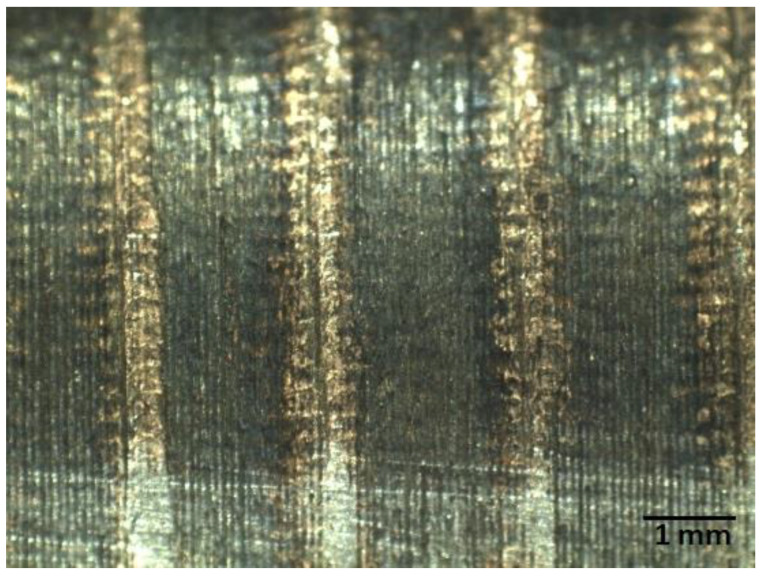
Regular microrelief with oil pockets to hold the grease in the process of friction—the study was carried out on a TM-505B microscope, Mitutoyo company, 40× steel, tenfold magnification.

A regular microrelief was obtained on the surface, with oil pockets to hold the grease during friction. During electromechanical processing (EMP), overlapping zones of the quenching bands appear, which depend on the feed rate S (Figure 2), with a decrease in hardness being observed in these zones. After EMP, a diamond smoother passing along the surface finally smooths out the microroughness of the profile, and when it reaches the overlapping zone, it forms grooves.

**Figure 3:**
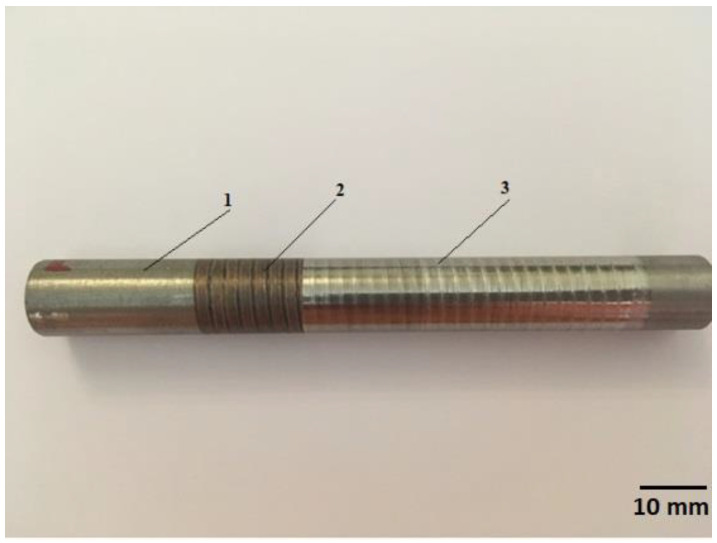
Samples for research: 1—area after machining, 2—area after electromechanical processing, 3—area after diamond smoothing.

For the study, we took samples made of steel grade 40× with a diameter of 12 ± 1 mm and a length of 120 ± 10 mm, processed with various modes of combined processing: electromechanical processing, with a current strength of 1000–2000 A, a voltage of 4 V, roll electrode pressure of 300 N, a part peripheral speed of 1.1–3.2 m/min, and a feed rate of 1–3.5 mm/rev; diamond smoothing, with a diamond smoother pressure of 200–250 N, a feed rate of 0.02 mm/rev, and a diamond smoother radius of 1 mm (Figure 3). The modes were determined earlier; this paper presents the ranges of modes in which a regular microrelief can be obtained. Twenty samples were taken for testing. There were 120 samples in total, and 20 were taken for this study (randomly). The value of residual stresses (Table 1) was considered as the average obtained at each stage of processing (1, 2, and 3 in Figure 3). At each stage, 5 measurements were taken.

To determine the phase composition of the samples and assess their structural state, the diffraction spectra were recorded on a Bruker D8 Discover diffractometer with the use of CuK_α_ radiation and a LynxEye position sensitive detector. The study was carried out in increments of 0.01° along the Bragg angle (2θ) and an accumulation of 1 s per detector strip, which in total gives about 170 s for each point in the angle. The phases were identified by means of Bruker AXS DIFFRAC.EVA v.4.2 software and the ICDD PDF-2 international database; TOPAS was used to determine the structural characteristics, and the X-ray reflection profile was adjusted using the data for a LaB6 reference sample (NIST SRM 660b). Macrostress measurements were performed using LEPTOS software.

We carried out the X-ray diffraction analysis of various areas of the surface layers of steel shafts: the initial state of the material was analyzed after lathe turning (LT), electromechanical processing (EMP), and diamond smoothing (DS). We took an X-ray spectrum for phase analysis of the material, assessed the structural state of the material after different types of processing along the X-ray line profile (310), and measured the residual macrostresses. In all processed areas, we took macrostress measurements which were carried out both in the axial (L) and tangential (T) directions.

Macrostresses were determined by the method of sample rotation [16,21,22,23,24,25]. The deformation of the interplanar spacings of the crystal structure of the samples’ surface layers was determined by the X-ray reflection shift (310). When calculating macrostresses, we used a Young’s modulus value of 181 GPa, as well as a Poisson’s ratio of 0.320. A large error in the measurement of macrostresses is determined by the unevenness of the studied surface of the processed samples areas. Nonetheless, it is possible to obtain regular changes in macrostresses depending on the type of processing.

## 3. Results

**Figure 4:**
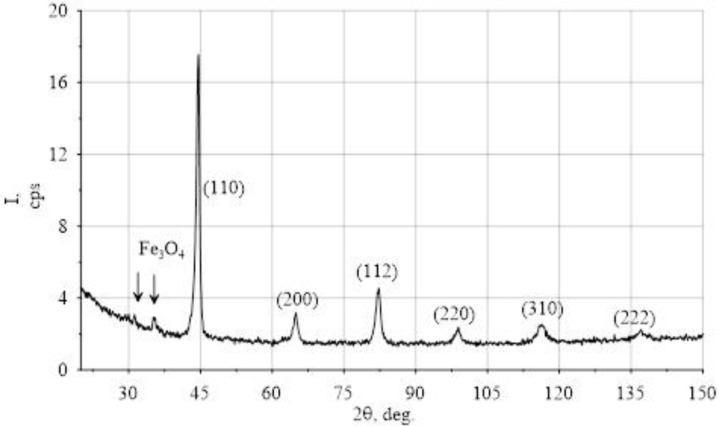
The diffraction spectrum of the surface layers of the shaft subjected to electromechanical processing (EMP). The figure shows the indices of X-ray reflections from the planes of the main phase (α-Fe). The arrows indicate the lines of the additional phase (iron oxide Fe_3_O_4_).

Figure 4 shows the diffraction spectrum of the sample area after EMP. According to the results obtained, there is an additional phase of iron oxide along with the main phase α-iron only in the areas subjected to electromechanical processing. The spectrum presented in Figure 4 shows the lines of this phase.

Table 1 shows the studies of macrostresses and structural characteristics at various stages of processing and the technological heredity effect on these values. Analyzing the obtained results, it can be assumed that diamond smoothing significantly increases the value of residual stresses in the surface layers due to an increase in the dislocation density. These findings coincide with the results in numerous works [2,3,6,7,8,13,16,21]. Since residual stresses significantly affect wear resistance, a reserve is created after combined processing to increase wear resistance.

Within electromechanical processing, residual stresses of different values and signs may be formed on the surface (Table 1). After diamond smoothing, however, all stresses become compressive. The value of residual stresses (Table 1) was considered as the average obtained at each stage of processing (1, 2, and 3 in Figure 3). At each stage, five measurements were taken.

**Figure 5:**
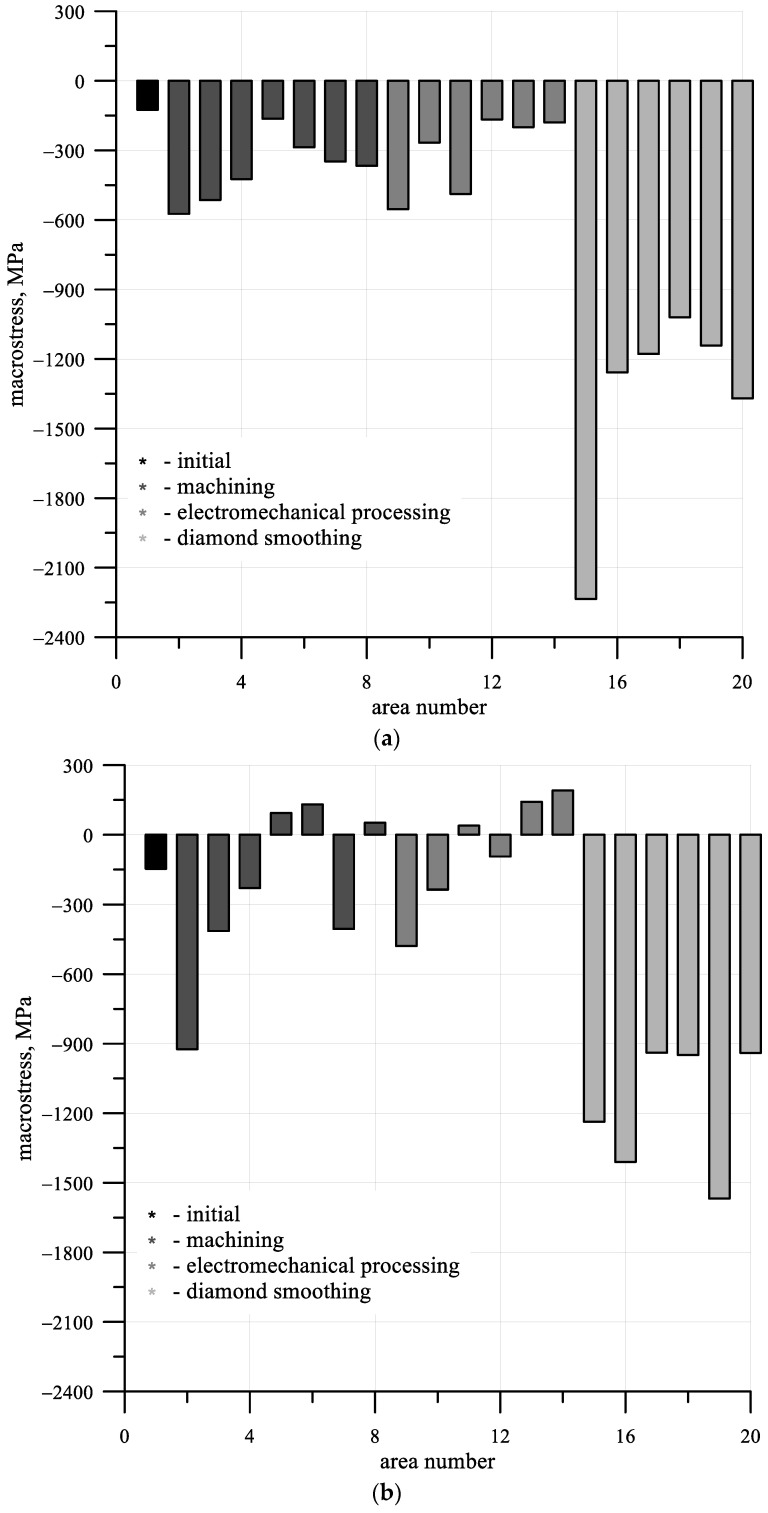
A graph of the dependence of axial (**a**) and circumferential (**b**) macrostresses value.

**Figure 6:**
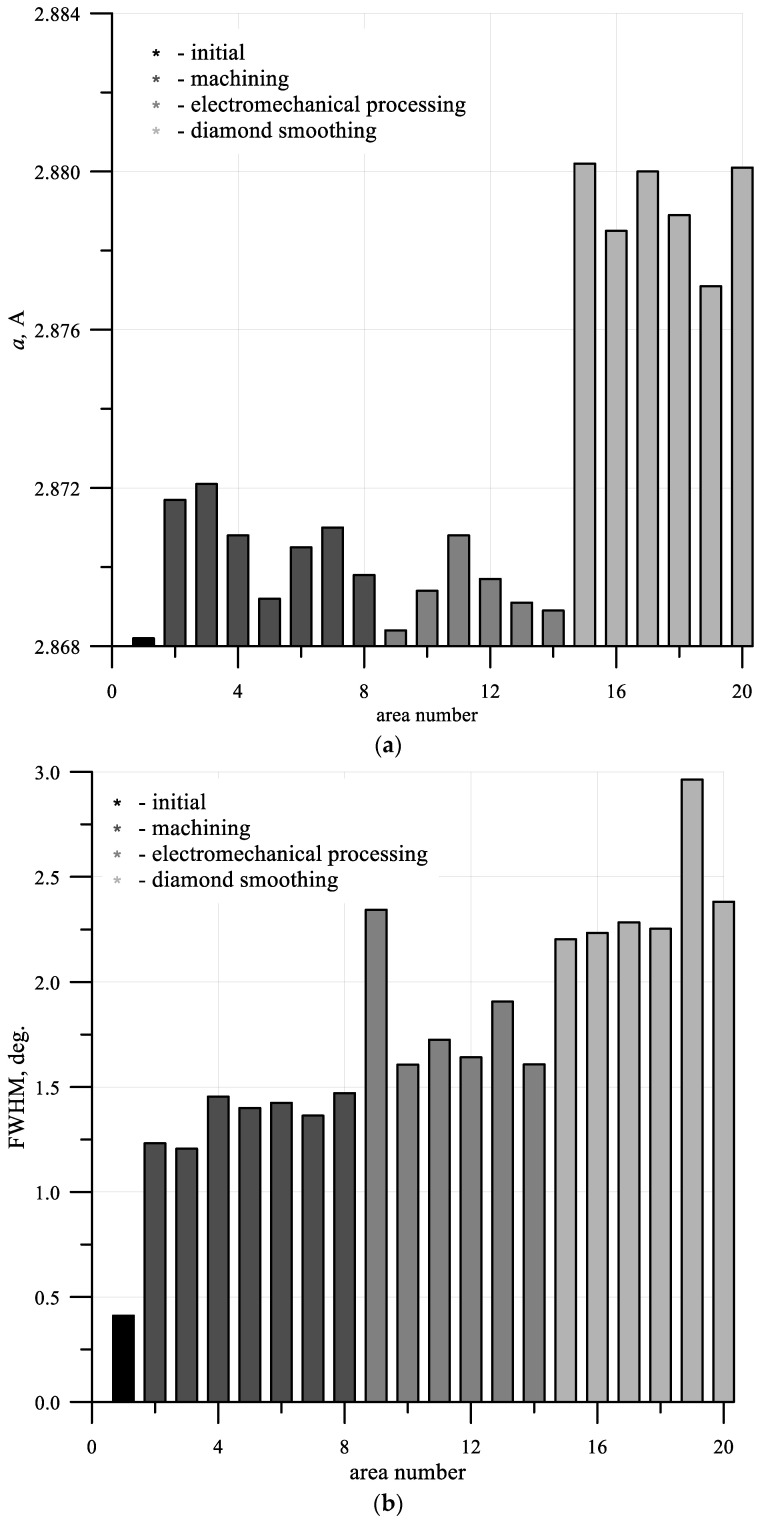
The dependence of the lattice parameter (**a**) and the half-width of the X-ray line (**b**).

Figure 5 shows graphs of the dependence of axial (a) and circumferential (b) residual macrostresses acting on different areas of the shafts under consideration. According to the results obtained, compressive residual macrostresses act on most of the investigated shaft areas, with rare exceptions. The maximum macrostress values can be observed in areas where diamond smoothing was carried out. For the same areas, the maximum width of X-ray reflections was recorded, indicating the maximum distortion of the crystal structure of the material (Figure 6b). In this case, the largest lattice spacing was also observed in areas processed with diamond smoothing (Figure 6a).

According to the data from the tables, the parameter of the crystal structure of α-iron is 2.8665 A. Carbon doping in accordance with the stable phase diagram leads to the formation of an additional phase in the steel–cementite. Taking into account the absence of carbon solubility in α-iron, we can consider the above parameter to be characteristic of the BCC-Fe of the steel we studied. All the measured parameters of the crystal structure of the α phase exceeded the reference value, which indicates the expansion of the crystal lattice in the radial direction of the shaft. Then, compressive residual stresses must act in the tangential and axial directions. The higher the lattice parameter value, the greater the activity of the compressive stresses in the axial and tangential directions.

The half-width of the X-ray line characterizes the fineness of the material structure and the value of the residual microstresses. The maximum values also correlate with the maximum compressive stresses.

## 4. Discussion

The purpose of this paper was to investigate the values of residual stresses in the surface layer.

As a result of the combined treatment, a regular microrelief is formed on the surface, and a hardened layer with high hardness is created. However, a high hardness does not always have a beneficial effect on performance indicators (e.g., on wear resistance). The combined treatment has proven its effectiveness: the hardness increases, the roughness decreases, and a regular microrelief is formed [17,18]. However, residual stresses also have a significant effect on wear resistance. Of interest is the result of how the residual stresses will change because of the action of two types of treatments: concentrated energy fluxes (EMP) and plastic deformation (diamond burnishing).

Because of the action of EMP, the hardness increases, and favorable residual stresses are created. After diamond blasting, the hardness is further increased. This is proved by measuring a, F in Table 1. Thus, a question may arise: Will the magnitude and sign of the residual stresses change? The sample measurements given in Table 1 prove that the residual stresses increase and remain compressive. The deformation of the crystal lattice indicates an increase in hardness on the sample’s surface, but there will be no fragility. This is consistent with previous tests [24,25].

Residual stresses were measured at all stages: after machining (turning), after processing with concentrated energy flows (EMP), and after plastic deformation (diamond burnishing).

This was done to analyze the hereditary influence of processing methods and modes on residual stresses in the surface layer. During the action of concentrated energy flows on the surface, a favorable microrelief (Figure 2), a favorable structure (fine-needle martensite) [19,20], and residual compressive stresses are created. These factors improve after diamond burnishing. This is a phenomenon of technological heredity. In addition, these factors will be present in the finished part and will have a positive effect on operational inheritance.

In the process of high-intensity processing, the surface layer of the samples absorbs a significant amount of energy in a short time, while nonequilibrium structures accumulating excess energy are formed in it. High-energy unstable structures themselves tend to a state with less free energy, which provides increased strength, wear resistance, and other operational characteristics of the surface layer.

## 5. Conclusions

These research results are associated with the phenomenon of technological heredity. The properties obtained at the first combined processing stage (EMP) have specific features—areas of overlapping hardening strips and a decrease in hardness in these areas. Diamond smoothing improves the surface quality: it reduces roughness, increases hardness, and a regular microrelief is obtained. This is the hereditary effect of combination machining modes on surface quality. Moreover, the surface quality is associated with wear resistance. Therefore, there is a relationship between the modes of combined processing with technological and operational heredity.

The findings of the research show that the value and sign of residual stresses depend on the modes of combined processing. According to the study, we recommend the following values: for electromechanical processing, a current strength of 1200–2000 A, a voltage of 4 V, a roll electrode pressure of 300 N, a part rotation speed of 1.1–2.0 m/min, and a feed rate of 1–1.8 mm/rev; for diamond smoothing, a diamond smoothing pressure of 200–250 N, a feed rate of 0.02 mm/rev, and a diamond smoothing radius of 1 mm.

The main parameters of the surface layer are formed at the final stage of the combined processing–diamond smoothing.

The main conclusions are as follows:

1. Studies of residual stresses after combined treatment have not been previously conducted.
2. This work shows the hereditary influence of the combined processing modes on the magnitude and sign of residual stresses.
3. The authors noted the transition of quantitative changes to qualitative ones: a combination of methods and modes of processing allows obtaining new properties on the surface with optimal values of residual stresses and for fatigue strength to be investigated.
4. The range of processing modes in which the research was carried out confirmed the correctness of the choice.
5. The authors plan to continue the research direction and check the adequacy of the theoretical model with practical results.
6. Combined processing can be recommended to harden the parts of power plants.

## Figures and Tables

**Table 1:** Macrostresses and structural characteristics of various areas of samples.

Type of Processing	Stresses, MPa		a, A	FWHM, Degrees
	Axial	Circumferential Stresses		
Machining	−125 ± 53	−147 ± 44	2.8682	0.4110
Electromechanical processing	−554 ± 15	−479 ± 406	2.8684	2.3448
Diamond smoothing	−2235 ± 119	−1237 ± 293	2.8802	2.2039
Diamond smoothing	−1142 ± 72	−1568 ± 354	2.8771	2.9638
Electromechanical processing	−180 ± 89	191 ± 131	2.8689	1.6080

a—Crystal lattice parameters; FWHM—full width at half maximum.
